# Brief Report: Real-World Comparative Effectiveness of First-Line Immune Checkpoint Inhibitor Monotherapy Versus Chemo-Immunotherapy in Metastatic NSCLC With PD-L1 More Than or Equal to 50%: A Meta-Analysis

**DOI:** 10.1016/j.jtocrr.2026.101011

**Published:** 2026-05-02

**Authors:** Thierry Landre, Jean-Baptiste Assié, Kader Chouahnia, Jean-Bernard Auliac, Gaëtan Des Guetz, Christos Chouaïd

**Affiliations:** a Hôpitaux Universitaires de Paris-Seine Saint-Denis, Hôpital René Muret, AP-HP, Sevran, France; b Department of Pneumology, Centre Hospitalier Intercommunal de Créteil, Créteil, France; c Université de Paris-Est Créteil, Créteil, France; d Functional Genomics of Solid Tumors Laboratory, Centre de Recherche des Cordeliers, Inserm, Sorbonne Université, Université Paris Cité, Paris, France; e Servie d'Oncologie, HUPSSD, Hôpital Avicenne, AP-HP, Bobigny, France; f Service d'Oncologie, Centre Hospitalier Delafontaine, Saint-Denis, France; g Inserm U955, UPEC, IMRB, Créteil, France

**Keywords:** PD-L1 high, NSCLC, Meta-analysis, Immunotherapy, Chemotherapy, First-line

## Abstract

**Introduction:**

For patients with metastatic NSCLC and high PD-L1 expression (≥50%), both immune checkpoint inhibitor (IO) monotherapy and chemo-immunotherapy (chemo-IO) are established first-line options. Although randomized trials have suggested similar overall survival (OS) between these strategies, real-world outcomes—especially in clinically heterogeneous populations—remain poorly defined.

**Methods:**

A systematic review and meta-analysis following Preferred Reporting Items for Systematic Reviews and Meta-Analyses 2020 guidelines was conducted. Eligible studies were retrospective comparative cohorts evaluating first-line IO monotherapy versus chemo-IO in adults with metastatic NSCLC and PD-L1 more than or equal to 50%. Primary outcome was OS; secondary outcomes included progression-free survival and objective response rate. Hazard ratios (HRs) were pooled using random-effects models.

**Results:**

A total of 19 retrospective studies comprising 11,048 patients were included (3337 chemo-IO; 7711 IO monotherapy). Chemo-IO significantly improved objective response rate (60% versus 41%). Pooled analyses demonstrated a benefit for chemo-IO in progression-free survival (HR = 0.75, 95% confidence interval [CI]: 0.67–0.85) and OS (HR = 0.82, 95% CI: 0.75–0.90). Subgroup analyses revealed marked heterogeneity: OS benefit of chemo-IO was pronounced in patients with Eastern Cooperative Oncology Group performance status of 0 (HR = 0.45, 95% CI: 0.32–0.63), in never-smokers (HR = 0.35, 95% CI: 0.17–0.70), and in women (HR = 0.68, 95% CI: 0.58–0.80). No significant OS advantage was observed in patients with PS more than or equal to 2 or age more than or equal to 75 years.

**Conclusions:**

In real-world practice, chemo-IO provides potential clinical benefit than IO monotherapy in PD-L1–high metastatic NSCLC, although the magnitude varies by patient characteristics. Ongoing prospective trials are essential to refine treatment selection and identify patients for whom chemotherapy can be safely omitted.

## Introduction

NSCLC constitutes the vast majority of bronchial malignancies, and approximately 30% of patients exhibit high tumor PD-L1 expression (≥50%).^1^ In this subgroup, the introduction of PD-1/PD-L1 inhibitors has profoundly reshaped first-line management. Phase III trials have demonstrated that single-agent immunotherapy with pembrolizumab,^2^ atezolizumab,^3^ or cemiplimab^4^ significantly improves overall survival (OS) and progression-free survival (PFS) compared with standard chemotherapy, establishing monotherapy as a therapeutic standard in tumors with high PD-L1 expression. In parallel, chemo-immunotherapy combinations—particularly those evaluated in KEYNOTE-189^5^^,^^6^ and KEYNOTE-407^7^^,^^8^—have demonstrated robust clinical benefit, regardless of PD-L1 expression level.

However, the relative superiority of monotherapy versus combination regimens in the setting of high PD-L1 expression remains intensely debated. Biologically, chemotherapy may reduce the risk of primary resistance to immunotherapy by modulating the tumor microenvironment, suggesting a potential advantage for combination strategies.^9^ Moreover, patients with a high tumor burden may derive particular benefit from a combined approach. Conversely, monotherapy minimizes toxicity, preserves quality of life, and is often favored in clinically fragile patients. Several indirect meta-analyses derived from randomized trials have compared these therapeutic strategies^10^, ^11^, ^12^: Immunotherapy plus chemotherapy was more effective than using immunotherapy alone as first-line therapy in terms of PFS and overall response rate (ORR). Nevertheless, there was no statistically significant difference observed between the two groups in terms of OS. To date, no meta-analysis has evaluated this question under real-world conditions, where patient characteristics, comorbidities, and treatment patterns differ substantially from those observed in the highly selected clinical trial populations.

This meta-analysis aims to address this gap and inform therapeutic decision-making in this critical subgroup of patients with metastatic NSCLC and PD-L1 more than or equal to 50%.

## Methods

### Search Strategy and Study Selection

A systematic review was conducted following Preferred Reporting Items for Systematic Reviews and Meta-Analyses 2020 guidelines. We searched PubMed/MEDLINE, the Cochrane Library, and the abstract databases of American Society of Clinical Oncology, European Society for Medical Oncology, and World Conference on Lung Cancer from inception to November 2025. Search terms included the following: “NSCLC,” “first-line,” “PD-L1 high expression,” “PD-L1 ≥ 50%,” “real-world,” “retrospective,” “immunotherapy monotherapy,” “chemo-immunotherapy,” and “combination therapy.” No publication date limits were applied. Titles, abstracts, and full texts were screened independently by two reviewers.

### Eligibility Criteria

We included retrospective real-world comparative cohort studies evaluating first-line systemic therapy in adults (≥18 y) with advanced or metastatic NSCLC and a tumor PD-L1 expression more than or equal to 50%. Eligible studies directly compared immune checkpoint inhibitor (IO) monotherapy—most often pembrolizumab—with chemo-immunotherapy (chemo-IO) consisting of a platinum-based doublet combined with an IO.

Studies were required to report at least one comparative outcome between treatment arms: OS, PFS, or objective response rate (ORR). Only observational real-world designs were eligible. Patients had to be free of targetable genomic alterations (*EGFR* mutations, *ALK* rearrangements, *ROS1* fusions). Studies including mixed PD-L1 populations were retained only when extractable results for the PD-L1 more than or equal to 50% subgroup were available.

We excluded randomized clinical trials, noncomparative series, reviews, commentaries, and studies in adjuvant, neoadjuvant, or locally advanced nonmetastatic settings. Special populations, SCLC, and data sets lacking stratified data for PD-L1–high tumors were also excluded. When overlapping data sets were identified, we retained the most complete or recent publication.

### Data Extraction

Two reviewers independently extracted study-level data using a predefined template, including the following: sample size per arm, median age, sex, Eastern Cooperative Oncology Group performance status (ECOG PS), smoking history, ORR, and hazard ratios (HRs) for OS and PFS with corresponding 95% confidence intervals (CIs). Discrepancies were resolved by consensus.

### Outcomes

The primary outcome was OS. Secondary outcomes included PFS and ORR. Prespecified subgroup analyses evaluated outcomes according to ECOG PS (0 versus 1 versus ≥2), smoking status (never versus former smokers), age (≥75 y versus <75 y), and sex (women versus men).

### Statistical Analysis

Random-effects models were applied to pool HRs for OS and PFS and risk ratios for ORR. Statistical heterogeneity was quantified using *I*^2^ statistics. Publication bias was assessed using funnel plots and Egger’s test when at least 10 studies contributed to an end point. All analyses were performed with Review Manager (RevMan) version 5.4.

## Results

**Figure 1:**
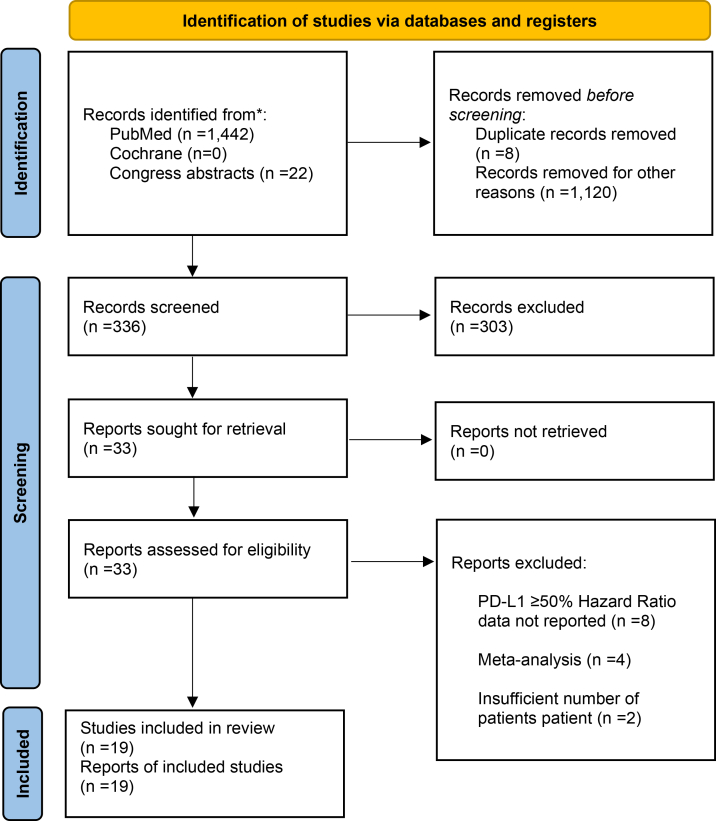
PRISMA flowchart summarizing the process for the identification of eligible studies. PRISMA, Preferred Reporting Items for Systematic Reviews and Meta-Analyses.

A total of 19 real-world retrospective studies (Fig. 1) (Supplementary Table 1) comprising a total of 11,048 patients with metastatic NSCLC and high PD-L1 expression (≥50%) were included. Nine studies specifically evaluated pembrolizumab, whereas ten assessed an unspecified PD-1/PD-L1 inhibitor (IO), administered either as monotherapy or in combination with platinum-based chemotherapy. Overall, 3337 patients received first-line chemo-IO and 7711 patients received IO monotherapy.

### Patients’ Characteristics

Baseline characteristics were broadly comparable across treatment groups. The mean age was slightly higher in the IO monotherapy cohort (weighted mean age 71 y) than in the chemo-IO cohort (67.4 y). The proportion of male patients was similar (66.4% versus 67.6%). Functional status differed modestly, with a higher proportion of ECOG PS 0 to 1 patient in the chemo-IO group (87.6%) than in the IO monotherapy group (80.8%). The percentage of never-smokers was low and balanced between arms (13.6% versus 12.3%). Nonsquamous histology predominated in both cohorts, with slightly higher representation in the chemo-IO group (74.3% versus 71.6%), although between-study heterogeneity was notable (Supplementary Table 1).

### Overall Response Rate, Progression-Free Survival, Overall Survival

**Figure 2:**
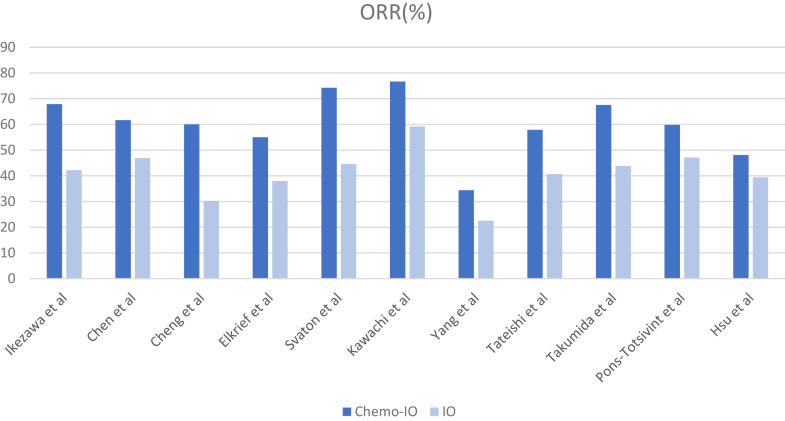
Meta-analysis of ORR. Chemo-IO, chemo-immunotherapy; IO, immune checkpoint inhibitor; ORR, overall response rate.

**Figure 3:**
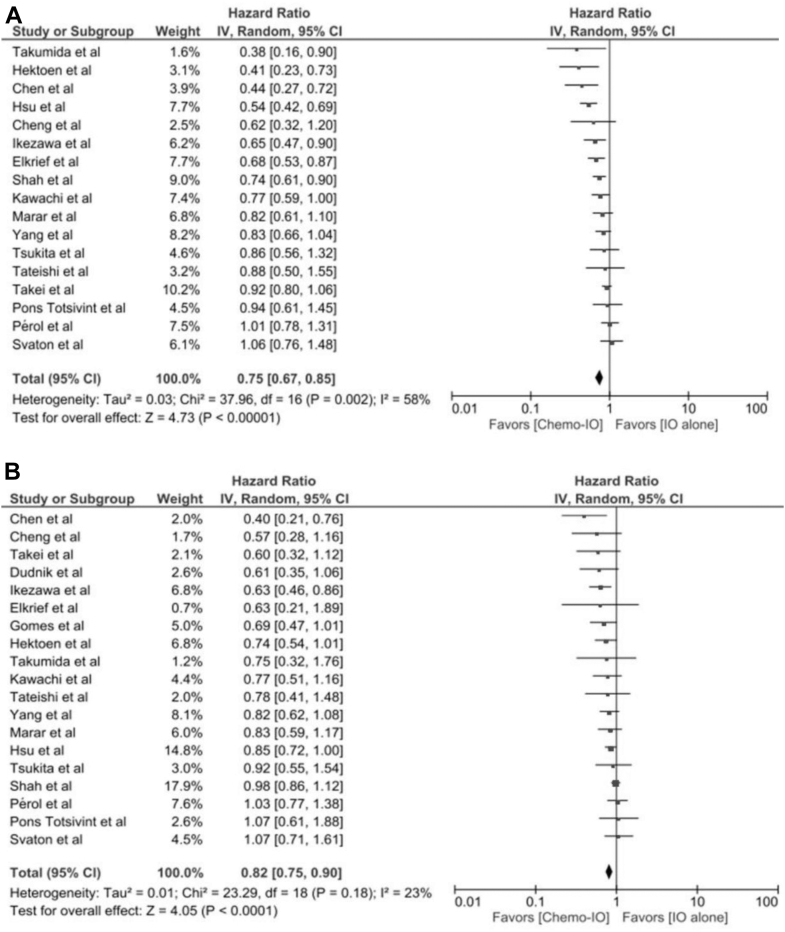
Meta-analysis of (*A*) progression-free survival and (*B*) overall survival. Chemo-IO, chemo-immunotherapy; CI, confidence interval; IO, immune checkpoint inhibitor.

Chemo-IO demonstrated superior antitumor activity, with a pooled ORR of 60% compared with 41% for IO monotherapy (Fig. 2). In survival analyses, chemo-IO was associated with a significant improvement in PFS (pooled PFS HR = 0.75, 95% CI: 0.67–0.85), corresponding to a 25% relative reduction in the risk of progression or death (Fig. 3*A*). OS was also significantly improved with chemo-IO (pooled OS HR = 0.82, 95% CI: 0.75–0.90), translating into an 18% reduction in mortality risk (Fig. 3*B*). Heterogeneity was low for OS (*I*^2^ = 23%) and moderate for PFS (*I*^2^ = 58%). These effects were consistent across studies evaluating pembrolizumab specifically and those assessing IO drugs as a class, despite variability in chemotherapy backbones and patient profiles.

### Subgroup Analyses of Overall Survival

For ECOG PS, OS favored chemo-IO in patients with PS of 0 (HR = 0.45, 95% CI: 0.32–0.64,*p* < 0.0001) and PS of 1 (HR = 0.86, 95% CI: 0.74–0.99, *p* = 0.004), but not in those with PS of 2 to 3 (HR = 0.95, 95% CI: 0.59–1.51; *p* = 0.81) (Supplementary Fig. 1). According to smoking status, chemo-IO significantly improved OS in never-smokers (HR = 0.35, 95% CI: 0.17–0.70, *p* = 0.003), whereas no benefit was observed in smokers (HR = 1.06, 95% CI: 0.83–1.36, *p* = 0.64) (Supplementary Fig. 2). Age-based analyses revealed that patients younger than 75 years derived significant benefit from chemo-IO (HR = 0.73, 95% CI: 0.58–0.91, *p* = 0.005), whereas no statistically significant advantage was observed in patients aged 75 years or older (HR = 0.79, 95% CI: 0.59–1.07, *p* = 0.13) (Supplementary Fig. 3). Regarding sex, women (HR = 0.68, 95% CI: 0.58–0.80, *p* < 0.0001) appeared to derive a greater OS benefit from chemo-IO than men (HR = 0.89, 95% CI: 0.77–1.02, *p* = 0.09) (Supplementary Fig. 4).

## Discussion

To our knowledge, this is the first meta-analysis of real-world data revealing the clinical benefit of chemo-IO in patients treated for NSCLC with PD-L1 expression more than or equal to 50%. However, the results should be interpreted with caution due to the numerous biases inherent in retrospective cohort data. To date, prospective evidence directly comparing pembrolizumab monotherapy with chemo-IO in PD-L1–high metastatic NSCLC has been limited. The PAULIEN trial,^13^ the first randomized head-to-head comparison of these two strategies, was prematurely stopped for futility and did not reveal a meaningful improvement in early tumor response, PFS, or OS with the addition of chemotherapy. Although its small sample size limits statistical power, PAULIEN suggests that the incremental added value of chemotherapy may be lower than expected in certain patient subsets.

Several ongoing randomized studies are expected to provide more definitive answers.^14^^,^^15^ The randomized, multicenter, phase III trial LAPLACE-50^15^ was limited to nonsquamous NSCLC PD-L1 more than or equal to 50% and compared the efficacy and safety of pembrolizumab with pembrolizumab-carboplatin-pemetrexed combination. It was a noninferiority study, with as primary end point, local PFS. The phase III trial PERSEE,^14^ a prospective, open-label, randomized study, randomized on a 1:1 basis chemo-IO or IO arms with a stratification according to tumor histology (squamous versus nonsquamous) and the presence or absence of brain metastasis. The primary objective is to evaluate the superiority of the chemo-IO combination compared with IO by evaluating PFS centrally reviewed. The recruitment was completed in the end of 2023, and results are planned for the end of 2026. Finally, the phase III INSIGNA trial (NCT03793179) evaluates whether pembrolizumab alone as a first-line treatment, followed by pemetrexed and carboplatin with or without pembrolizumab after disease progression, is superior to induction with pembrolizumab, pemetrexed, and carboplatin followed by pembrolizumab and pemetrexed maintenance in treating patients with stage IV NSCLC PD-L1 more than or equal to 50%.

In all cases, the interpretation of these results must be cautious as all included studies are retrospective and nonrandomized, with baseline imbalances in real-world OS differences may reflect patient selection and treatment allocation patterns rather than true superiority of chemo-IO in PD-L1–high disease. Moreover, OS in real-world data sets is strongly influenced by access to and sequencing of subsequent lines of therapy, which are not accounted for in this meta-analysis Similarly, subgroup analyses should be considered as hypothesis generating rather than clinically directive, as there may be biases related to residual confounding and multiple testing.

## Conclusion

Although it is necessary to wait the results of ongoing randomized trials, this meta-analysis on real-world practice data reveals a potential OS benefit of chemo-IO combination in metastatic NSCLC with a PD-L1 more than or equal to 50%, although the magnitude varies by patient characteristics.

## CRediT Authorship Contribution Statement

**Thierry Landre**: Conceptualization, Methodology, Data curation, Project administration, Writing - original draft, Writing - review & editing.

**Jean-Baptiste Assié**: Conceptualization, Methodology, Data curation, Writing - original draft, Writing - review & editing.

**Kader Chouahnia**: Conceptualization, Writing - review & editing.

**Jean-Bernard Auliac**: Supervision, Writing - review & editing.

**Gaëtan Des Guetz**: Supervision, Writing - review & editing.

**Christos Chouaïd**: Project administration, Supervision, Writing - original draft, Writing - review & editing.

## Disclosure

Dr. Chouaïd declares having received grants, travel expenses, and consulting fees from AZ, BI, GSK, Roche, Sanofi Aventis, BMS, MSD, Lilly, Novartis, Pfizer, Takeda, Bayer, Janssen, and Amgen. Dr. Assié declares having received fees from AZ, MSD, and BMS. Dr. Auliac declares having received fees from AZ, BI, Sanofi Aventis, BMS, MSD, Lilly, Takeda, and Janssen. The remaining authors declare no conflict of interest.
