# Supplementary material for: Brief Report: Real-World Comparative Effectiveness of First-Line Immune Checkpoint Inhibitor Monotherapy Versus Chemo-Immunotherapy in Metastatic NSCLC With PD-L1 More Than or Equal to 50%: A Meta-Analysis
Source: JTO Clin Res Rep. 2026 May 2;7(7):101011. doi: 10.1016/j.jtocrr.2026.101011 (PMC13253113; doi:10.1016/j.jtocrr.2026.101011)
Supplement: Supplement Data [file mmc1.docx]

**Supplementary Data:**

**Supplementary Figure 1:** Meta-analysis of Overall Survival (OS) based on ECOG Performance Status (PS)

**Supplementary Figure 2:** Meta-analysis of Overall Survival (OS) based on Smoking Status

**Supplementary Figure 3:** Meta-analysis of Overall Survival (OS) based on Age (Younger *versus* Older 75 years)

**Supplementary Figure 4:** Meta-analysis of Overall Survival (OS) based on Sex patient’s

**Supplementary Table 1:** Patient characteristics of the 19 studies included in the meta-analysis. References of the 19 studies included in the meta-analysis.

**Supplementary Figure 1:** Meta-analysis of Overall Survival (OS) based on ECOG Performance Status (PS)


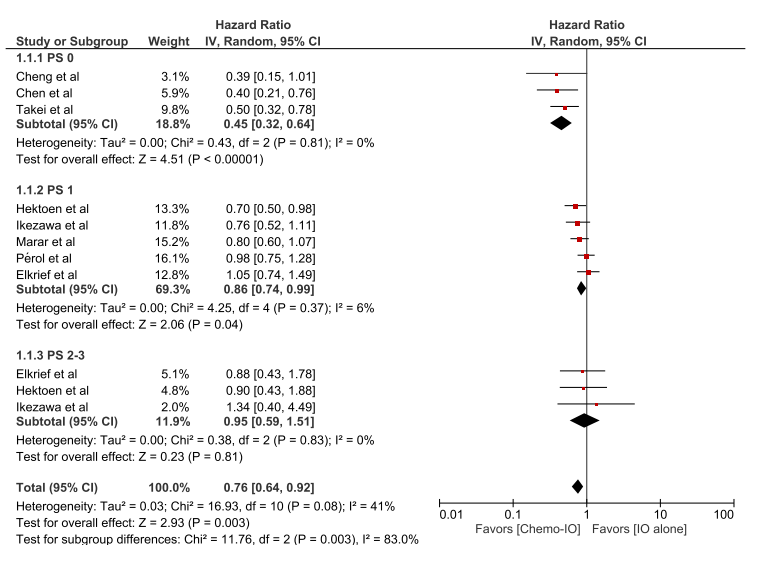


**Supplementary Figure 2:** Meta-analysis of Overall Survival (OS) based on Smoking Status


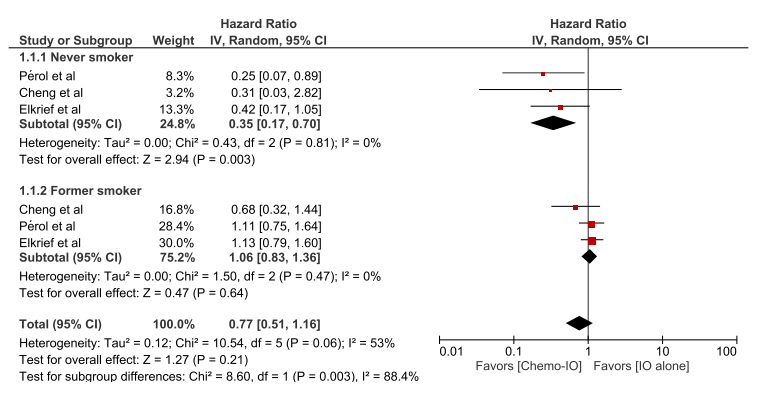


**Supplementary Figure 3:** Meta-analysis of Overall Survival (OS) based on Age (Younger *versus* Older 75 years)


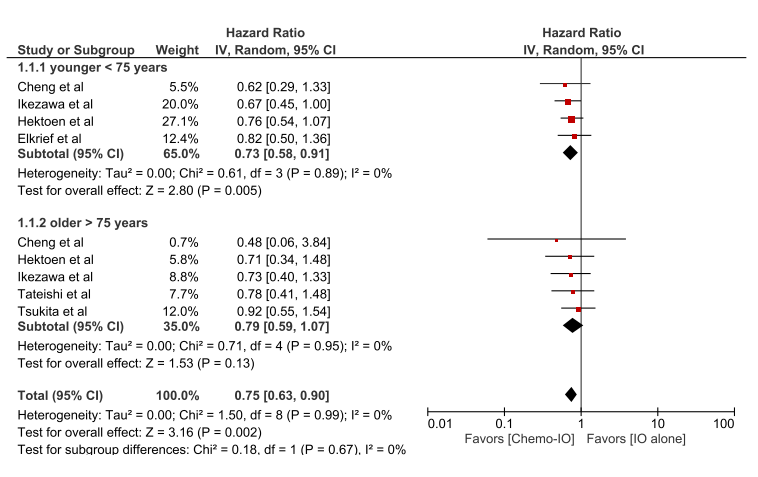


**Supplementary Figure 4:** Meta-analysis of Overall Survival (OS) based on Sex patient’s


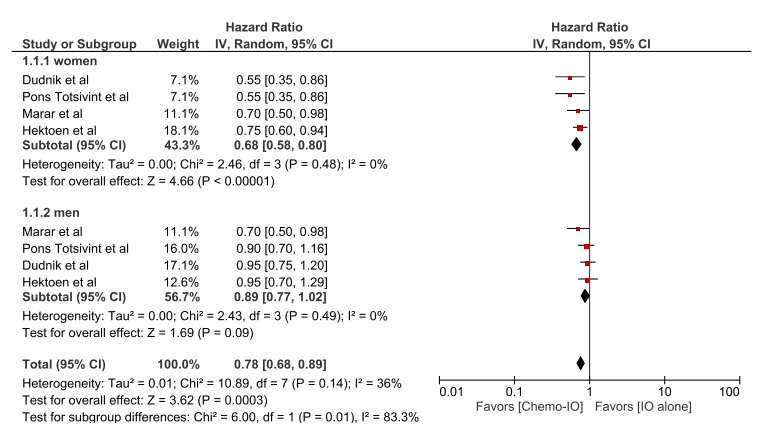


**Supplementary Table 1:** Main characteristics of the 19 studies included in the meta-analysis

| **First author** | **Country** | **Study objective** | **Immunotherapy used** | **HR for OS** |
| --- | --- | --- | --- | --- |
| *Hektoen et al.*(1) | Norway | Compare pembrolizumab vs pembrolizumab + chemo in PD-L1 ≥50% adenocarcinoma | Pembrolizumab ± chemotherapy | adjusted |
| *Dudnik et al.*(2) | Israel | Real-world comparison in PD-L1 ≥50% and ≥90% | Pembrolizumab ± chemotherapy | adjusted |
| *Svaton et al.*(3) | Czech Republic | Multicenter comparison pembrolizumab vs pembrolizumab + chemo | Pembrolizumab ± chemotherapy | undajusted |
| *Pérol et al.*(4) | Europe | Real-world effectiveness IO vs chemo-IO in PD-L1-high non-squamous NSCLC | PD-(L)1 inhibitors ± chemotherapy | adjusted |
| *Shah et al.*(5) | USA | Real-world-effectiveness PD-L1 ≥50% and ≥90% | Pembrolizumab ± chemotherapy | adjusted |
| *Elkrief et al.*(6) | USA/Canada | IO vs IO + chemotherapy in PD-L1+ adenocarcinoma | PD-(L)1 inhibitors ± chemotherapy | adjusted |
| *Cheng et al.*(7) | Australia | Real-world survival comparison in PD-L1-high NSCLC | IO (not specified) ± chemotherapy | undajusted |
| *Takei et al.*(8) | Japan | Older adults PD-L1 ≥50% treated with IO ± chemo | IO ± chemotherapy | adjusted |
| *Ikezawa et al.*(9) | Japan | Real-world outcomes of 1L pembrolizumab | Pembrolizumab ± chemotherapy | unadjusted |
| *Tateishi et al.*(10) | Japan | Elderly subgroup analysis of pembrolizumab | Pembrolizumab ± chemotherapy | unadjusted |
| *Kawachi et al.*(11) | Japan | Impact of PPIs with pembrolizumab vs chemo-IO | Pembrolizumab ± chemotherapy | adjusted |
| *Marar et al.*(12) | USA | National Real-world-effectiveness PD-L1-high patterns and outcomes | PD-L1 inhibitors ± chemotherapy | adjusted |
| *Yang et al.*(13) | China | Ki-67 biomarker stratification | IO ± chemotherapy | unadjusted |
| *Hsu et al.*(14) | USA | Real-world registry (abstract) | PD-(L)1 inhibitors ± chemotherapy | adjusted |
| *Gomes et al.*(15) | UK | NCRAS registry PD-L1-stratified stage IV NSCLC | IO ± chemotherapy | unadjusted |
| *Takumida et al.*(16) | Japan | Time-to-failure pembrolizumab vs pembrolizumab + chemo | Pembrolizumab ± chemotherapy | adjusted |
| *Tsukita et al.*(17) | Japan | Older adults receiving IO vs chemo-IO | IO ± chemotherapy | adjusted |
| *Pons-Tostivint et al.*(18) | France | Multicenter Real-world-effectiveness pembrolizumab ± chemo PD-L1 ≥50% | Pembrolizumab ± chemotherapy | adjusted |
| *Chen et al.*(19) | China | Retrospective pembrolizumab ± chemo PD-L1 ≥50% | Pembrolizumab ± chemotherapy | unadjusted |

IO: Immunotherapy; Chemo: chemotherapy; 1L: First Line; PPIs: Proton Pump Inhibitors; NCRAS: National Cancer Registration Analysis Service; NSCLC: Non-Small Cell Lung Cancer; HR: Hazard ratio; OS: Overall Survival

**Supplementary Table 1 (continuation):** Patient characteristics of the 19 studies included in the meta-analysis

| **Author** | **N** | | **Median age (years)** | | **Sexe male (%)** | | **PS 0-1 (%)** | | **Never smoker (%)** | | **Non-squamous (%)** | |
| --- | --- | --- | --- | --- | --- | --- | --- | --- | --- | --- | --- | --- |
|  | **IO** | **Chemo-IO** | **IO** | **Chemo-IO** | **IO** | **Chemo-IO** | **IO** | **Chemo-IO** | **IO** | **Chemo-IO** | **IO** | **Chemo-IO** |
| *Ikezawa et al* | 166 | 134 | 74 | 68 | 79 | 79 | 70 | 92 | 15 | 12 | 55 | 59 |
| *Chen et al* | 91 | 115 | 67 | 65 | 88 | 88 | 100 | 100 | 21 | 25 | 54 | 64 |
| *Cheng et al* | 86 | 25 | 71 | 65 | 76 | 76 | 82 | 86 | 6 | 12 | 81 | 88 |
| *Dudnik et al* | 203 | 53 | 68 | 64 | 68 | 58 | 68 | 85 | 8 | 9 | 78 | 72 |
| *Hektoen et al* | 317 | 93 | 69 | 66 | 46 | 45 | 75 | 88 | NA | NA | 100 | 100 |
| *Tsukita et al* | 297 | 75 | 77 | 77 | 88 | 88 | 84 | 84 | 9 | 9 | 60 | 60 |
| *Elkrief et al* | 398 | 117 | 69 | 67 | 42 | 46 | 84 | 86 | 8 | 16 | 100 | 100 |
| *Svaton et al* | 706 | 87 | 69 | 69 | 60 | 66 | 98 | 92 | 9 | 11 | 58 | 78 |
| *Pérol et al* | 351 | 169 | 72 | 67 | 48 | 56 | 100 | 100 | 10 | 9 | 100 | 100 |
| *Kawachi et al* | 131 | 131 | NA | NA | NA | NA | NA | NA | NA | NA | NA | NA |
| *Shah et al* | 2098 | 988 | 73 | 68 | 50 | 56 | 55 | 62 | 6 | 7 | 71 | 78 |
| *Pons-Totsivint et al* | 141 | 102 | 68 | 61 | 58 | 56 | 75 | 82 | 8 | 3 | 82 | 95 |
| *Marar et al* | 178 | 133 | 73 | 67 | 51 | 57 | 66 | 71 | 9 | 7 | 79 | 80 |
| *Yang et al* | 157 | 177 | 62 | 62 | 75 | 75 | 88 | 88 | 33 | 33 | 55 | 55 |
| *Tateishi et al* | 81 | 19 | 79 | 76 | 80 | 89 | 75 | 100 | 18 | 16 | 58 | 31 |
| *Takumida et al* | 89 | 37 | 69 | 64 | 73 | 73 | 73 | 86 | 15 | 15 | 57 | 70 |
| *Hsu et al* | 900 | 484 | NA | NA | NA | NA | NA | NA | NA | NA | NA | NA |
| *Gomes et al* | 1190 | 330 | NA | NA | NA | NA | NA | NA | NA | NA | NA | NA |
| *Takei et al* | 131 | 68 | 76 | 73 | 81 | 73 | 100 | 100 | 10 | 20 | 58 | 58 |
| **Total** | **7711** | **3337** | **71** | **67,4** | **66,4** | **67,5** | **80,8** | **87,6** | **12,3** | **13,6** | **71,6** | **74,2** |

IO: Immunotherapy; Chemo: chemotherapy, N: number of patients; PS: Performance status

**References of the 19 studies included in the meta-analysis**

1. Hektoen HH, Tsuruda KM, Brustugun OT, Neumann K, Andreassen BK. Real-world comparison of pembrolizumab alone and combined with chemotherapy in metastatic lung adenocarcinoma patients with PD-L1 expression ≥50. ESMO Open. mai 2025;10(5):105073.

2. Dudnik E, Moskovitz M, Rottenberg Y, Lobachov A, Mandelboim R, Shochat T, et al. Pembrolizumab as a monotherapy or in combination with platinum-based chemotherapy in advanced non-small cell lung cancer with PD-L1 tumor proportion score (TPS) ≥50%: real-world data. Oncoimmunology. 28 janv 2021;10(1):1865653.

3. Svaton M, Knetki-Wroblewska M, Hosek P, Chowaniecova G, Spacek J, Fischer O, et al. Pembrolizumab versus Pembrolizumab plus Chemotherapy in Non-small Cell Lung Cancer with High PD-L1 Expression - Multicenter Real-world Evidence Study. J Cancer. 2025;16(10):3015‑23.

4. Pérol M, Felip E, Dafni U, Polito L, Pal N, Tsourti Z, et al. Effectiveness of PD-(L)1 inhibitors alone or in combination with platinum-doublet chemotherapy in first-line (1L) non-squamous non-small-cell lung cancer (Nsq-NSCLC) with PD-L1-high expression using real-world data. Ann Oncol Off J Eur Soc Med Oncol. mai 2022;33(5):511‑21.

5. Shah M, Mamtani R, Marmarelis ME, Hennessy S. Chemoimmunotherapy vs. Immunotherapy for First Line Treatment of Advanced Non-small Cell Lung Cancer With a PD-L1 Expression ≥50% or ≥90. Clin Lung Cancer. mai 2023;24(3):235‑43.

6. Elkrief A, Alessi JMV, Ricciuti B, Brown S, Rizvi H, Preeshagul IR, et al. Efficacy of PD-(L)1 blockade monotherapy compared with PD-(L)1 blockade plus chemotherapy in first-line PD-L1-positive advanced lung adenocarcinomas: a cohort study. J Immunother Cancer. juill 2023;11(7):e006994.

7. Cheng J, McKay C, Bray V, Yip PY, Tognela A, Kok PS. Real-World Survival Outcomes of Patients With High PD-L1 Advanced NSCLC Who Received Chemoimmunotherapy Versus Immunotherapy. Asia Pac J Clin Oncol. 17 juin 2025;e14205.

8. Takei S, Kawachi H, Yamada T, Tamiya M, Negi Y, Goto Y, et al. Prognostic impact of clinical factors for immune checkpoint inhibitor with or without chemotherapy in older patients with non-small cell lung cancer and PD-L1 TPS ≥ 50%. Front Immunol. 23 févr 2024;15:1348034.

9. Ikezawa Y, Mizugaki H, Morita R, Tateishi K, Yokoo K, Sumi T, et al. Current status of first-line treatment with pembrolizumab for non-small-cell lung cancer with high PD-L1 expression. Cancer Sci. juin 2022;113(6):2109‑17.

10. Tateishi K, Mizugaki H, Ikezawa Y, Morita R, Yokoo K, Sumi T, et al. Real-world data of first-line treatment with pembrolizumab for NSCLC with high PD-L1 expression in elderly patients: a subgroup analysis of HOT/NJLCG2001. Jpn J Clin Oncol. 5 mars 2025;55(3):253‑60.

11. Kawachi H, Yamada T, Tamiya M, Negi Y, Goto Y, Nakao A, et al. Concomitant Proton Pump Inhibitor Use With Pembrolizumab Monotherapy vs Immune Checkpoint Inhibitor Plus Chemotherapy in Patients With Non-Small Cell Lung Cancer. JAMA Netw Open. 3 juill 2023;6(7):e2322915.

12. Marar R, Bai C, Hansen E, Zettler CM, Belli AJ, Fernandes LL, et al. Treatment Patterns and Outcomes of Non-Small Cell Lung Cancer with High PD-L1 Expression using Real World Evidence. Clin Lung Cancer. nov 2025;26(7):564-572.e4.

13. Yang N, Chen X, Wang H, Liu P, Liu B, Xie B, et al. Ki-67 expression stratifies PD-L1-high NSCLC for immune checkpoint inhibitor plus chemotherapy: a real-world biomarker validation. Cancer Immunol Immunother CII. 3 nov 2025;74(12):354.

14. Hsu ML, Xia Q, Aggarwal S, Nkhoma E, R D, Hager T, et al. EP.11A.08 Real-World Comparative Effectiveness in Advanced NSCLC and High PD-L1 with 1L Immune Checkpoint Inhibitors ± Chemotherapy. J Thorac Oncol. oct 2024;19(10):S598.

15. Gomes F, Lee H, Ingleby F, Bouajila I, Sudhapalli P, Chaparova E, et al. 1355P Real-world (RW) treatment patterns and survival outcomes by PD-L1 expression in stage IV non-squamous (nsq) non-small cell lung cancer (NSCLC): A retrospective analysis of the UK National Cancer Registry Database (NCRAS). Ann Oncol. sept 2024;35:S855.

16. Takumida H, Horinouchi H, Masuda K, Shinno Y, Okuma Y, Yoshida T, et al. Comparison of time to failure of pembrolizumab plus chemotherapy versus pembrolizumab monotherapy: a consecutive analysis of patients having NSCLC with high PD-L1 expression. Cancer Immunol Immunother CII. mars 2022;71(3):737‑46.

17. Tsukita Y, Tozuka T, Kushiro K, Hosokawa S, Sumi T, Uematsu M, et al. Immunotherapy or Chemoimmunotherapy in Older Adults With Advanced Non-Small Cell Lung Cancer. JAMA Oncol. 1 avr 2024;10(4):439‑47.

18. Pons-Tostivint E, Hulo P, Guardiolle V, Bodot L, Rabeau A, Porte M, et al. Real-world multicentre cohort of first-line pembrolizumab alone or in combination with platinum-based chemotherapy in non-small cell lung cancer PD-L1 ≥ 50. Cancer Immunol Immunother CII. juin 2023;72(6):1881‑90.

19. Chen Y, Wang Y, Yang Z, Hu M, Zhang Y, Qian F, et al. Pembrolizumab Alone or Combined With Chemotherapy in Advanced NSCLC With PD-L1 ≥50%: Results of a Retrospective Study. Front Oncol. 2021;11:691519.
